# Insufficiency of annual praziquantel treatment to control *Schistosoma mansoni* infections in adult women: A longitudinal cohort study in rural Tanzania

**DOI:** 10.1371/journal.pntd.0007844

**Published:** 2019-11-21

**Authors:** Pallavi Mishra, Soledad Colombe, Ndalloh Paul, Jane Mlingi, Inobena Tosiri, Christine Aristide, Joanna Gao, Philibert Kashangaki, Honest Nagai, Samuel E. Kalluvya, Claudia J. de Dood, Paul L. Corstjens, Julius Mngara, Govert J. van Dam, Jennifer A. Downs

**Affiliations:** 1 Center for Global Health, Department of Medicine, Weill Cornell Medicine, New York, NY, United States of America; 2 Bugando Medical Centre, Mwanza, Tanzania; 3 National Institute for Medical Research, Mwanza, Tanzania; 4 Department of Medicine, Catholic University of Health and Allied Sciences, Mwanza, Tanzania; 5 Department of Cell and Chemical Biology, Leiden University Medical Center, Leiden, Netherlands; 6 Department of Parasitology, Leiden University Medical Center, Leiden, Netherlands; RTI International, UNITED STATES

## Abstract

**Background:**

Current World Health Organization (WHO) guidelines recommend annual mass drug administration using praziquantel in areas with high schistosome endemicity. Yet little is known about incidence and reinfection rates after treatment in women with frequent exposure to schistosomes. We sought to quantify response to anti-schistosome treatment and incident *S*. *mansoni* infections in a cohort of rural women living in a schistosome-endemic area of northwest Tanzania.

**Methods and principal findings:**

We enrolled women with and without *S*. *mansoni* infection into a 12-month longitudinal cohort. Every 3 months, women were tested for schistosome infection using microscopic examinations for ova on filtered urine, Kato Katz slides, and serum Circulating Anodic Antigen (CAA). Those with schistosome infection received treatment with praziquantel 40 mg/kg according to the standard of care.

We studied 35 women who were *S*. *mansoni* positive by stool microscopy and 46 women without schistosome infection who returned for at least one follow-up. Of the women who were initially infected, 14 (40%) were schistosome-positive at a follow-up visit. Four women developed incident infections, for a cumulative incidence of 8.7% and incidence rate of 0.99 per 100 person-months throughout the year among initially uninfected women. Only 3 women were egg-positive at any follow-up.

Women with persistent, recurrent, or incident infection during the study period were significantly younger (p = 0.032) and had fewer children than women who remained uninfected or those who cleared the infection and did not experience recurrence (p = 0.003). Having fewer children remained significant after controlling for age (p = 0.023). There was no difference in initial intensity of infection by CAA or stool egg count, HIV status, or socioeconomic status. Although most water contact behaviors were comparable between the two groups, women with recurrent or incident schistosome infections were significantly more likely to have recently swum in the lake (p = 0.023).

**Conclusions:**

Our data suggests that annual praziquantel treatment reduces intensity of schistosome infections but is insufficient in providing stable parasite eradication in over a third of women in endemic communities. Furthermore, microscopy lacks adequate sensitivity to evaluate efficacy of treatment in this population. Our work demonstrates that further investigation into treatment efficacy and reinfection rates is warranted and suggests that increased frequency of praziquantel treatment is needed to improve cure rates in high-risk populations.

## Author summary

Schistosomiasis is a parasitic infection transmitted through contaminated water that primarily affects the gastrointestinal and urogenital tracts. Previous studies in Tanzania have shown that adult women infected with schistosomes also have a higher risk of contracting HIV. Although it is recommended that people living in areas where they are exposed to schistosomes be treated with praziquantel once a year, the rate of new infections or reinfection after treatment in adult women is not known. We followed a group of schistosome-infected women and an uninfected control group for 12 months. They were tested for schistosomes every 3 months, and treated with praziquantel if they were infected. Over 40% of the women tested positive for schistosome infection at some point during the follow-up period, and the majority of them were from the group that was infected at the beginning of the study. These women may not have fully cleared the infection after one treatment, or they may be more susceptible to reinfection due to variations in their immune systems. Further studies are recommended to investigate whether a higher frequency of treatment is needed to control schistosome infection in adult women, especially given that reducing schistosome infection may help to reduce HIV risk in populations similar to ours.

## Introduction

Schistosomiasis is a water-borne helminthic infection, most prominently affecting the urogenital and gastrointestinal tracts, that causes disease in over 200 million people worldwide [1]. Schistosomes have a complex life cycle that includes freshwater snails as intermediate hosts, from which infective cercariae are released into fresh water and can penetrate unbroken skin. The disease is highly prevalent in tropical climates where the parasite is common and access to clean water is limited. Over 90% of people with schistosome infections live in Africa [1], and the Lake Victoria region in Tanzania has one of the highest prevalences of schistosome infections in the world [2,3].

World Health Organization guidelines recommend annual mass treatment with praziquantel 40 mg/kg in schistosome-endemic regions [4]. Although recent studies have suggested higher cure rates with repeated dosing or single doses of 60 mg/kg, a single dose of 40 mg/kg remains the mainstay of most schistosome control programmes [5] and is the standard of care in Tanzania. Moreover, these recommendations are largely based on studies in school-age or younger children, and little is known about incidence and reinfection rates after treatment in adults. In rural communities where women’s responsibilities to collect water, cook and wash cause them to contact contaminated water on a daily basis, more than half of women have schistosome infections [3,6,7]. Furthermore, recent studies in Tanzania and Zambia have shown that infection with *Schistosoma mansoni* increases susceptibility to HIV infection in women, but not in men, and raises HIV-1 RNA viral load set point in those who become infected [8,9]. If incidence and reinfection rates among women are high, then recommendations for yearly anti-schistosome treatment may need to be increased. This could serve not only to decrease the morbidity and mortality related to schistosome infection itself and to improve population-level control of schistosomiasis, but additionally to decrease the HIV risk that accompanies schistosome infection in women.

We hypothesized that the overall incidence of schistosome infections in reproductive-aged women would be at least 6% at 6 months and 12% over the course of the year, including women who were schistosome-infected at baseline and those who were not. We based these hypotheses on 10 years of clinical experience in this region. We also predicted that at least 20% of women treated for schistosomiasis would show evidence of incomplete clearance with standard of care single-dose praziquantel [10,11]. Secondary hypotheses were that incident schistosome infections would be associated with age, water security, and the intensity of initial schistosome infection. Our overall objective was to determine the clearance, recurrence, and incidence of *S*. *mansoni* infection in a population of women of childbearing age living in rural northwest Tanzania in the year after treatment with praziquantel.

## Materials and methods

### Ethics

Women provided written informed consent, obtained by the nurse in a private setting in Kiswahili, for study participation. All participants were adults. All treatment was provided free of charge. Any woman who tested positive for HIV on an initial point-of-care test (SD Bioline, Standard Diagnostics, Inc., Korea) underwent confirmatory testing using a second point-of-care test (Uni-gold, Trinity Biotech, Wicklow, Ireland) in accordance with Tanzanian national guidelines. HIV test results were provided on the day of screening, and any woman with a new diagnosis of HIV was counselled and provided with a referral letter to obtain free treatment at a nearby HIV care and treatment center.

Permission for the conduct of this study was obtained from the joint research ethics committee of Bugando Medical Centre/the Catholic University for Health and Allied Sciences in Mwanza, Tanzania (CREC/171/2017), from the National Institute for Medical Research in Dar es Salaam (NIMR/HQ/R.8a/Vol.IX/2446), and from Weill Cornell Medicine in New York (1612017800).

### Study population and design

We conducted a longitudinal study of women with and without *S*. *mansoni* infection in villages in rural Tanzania in which the prevalence *of S*. *mansoni* infection in adults is known to be approximately 40% and the prevalence of *S*. *haematobium* infection is < 2% [8]. Enrollment was conducted between June and November 2017 in the schistosome-endemic villages of Kisesa, Lumeji, Welamasonga, and Kayenze. Women of reproductive age living in these villages were invited to receive free screening for schistosomiasis via urine, stool, and blood tests. Women who had schistosome infections were treated with praziquantel free of charge, and all women were asked to return after 3, 6, 9, and 12 months for repeat testing and examinations.

This analysis was conducted as part of a larger cohort study to characterize alterations induced by *S*. *mansoni* infection in the female genital tract and to determine the effect of anti-schistosome praziquantel treatment on these alterations. In order to study the dynamics of *S*. *mansoni* infection and praziquantel treatment, we restricted our analysis to women who returned for at least one of the four follow-up visits.

### Sample collection and laboratory procedures

At screening, women provided urine, stool, and blood to be tested for schistosome infection. Five Kato Katz slides were prepared from each stool sample for examination for *S*. *mansoni* ova, a technique that maximizes sensitivity without the need for stool examinations on multiple days [12]. 10 mL of urine was filtered from each sample and examined microscopically in the field for *S*. *haematobium* ova by trained parasitologists.

A three milliliter sample of blood was also collected for quantification of serum CAA, which was performed using up-converting phosphor technology at the Tanzanian National Institute for Medical Research laboratory in Mwanza as previously described [13]. We defined a negative CAA value as < 30 pg/mL [13].

Women were given cards with a follow-up date within 1–2 weeks to receive their urine, stool, and serum CAA results verbally and in writing, and to receive directly observed treatment with praziquantel if any test was positive. At that time, we invited those who were positive for *S*. *mansoni* infection to participate in our cohort. Although we expected Kato Katz sensitivity to be lower than CAA testing in these women [14], we required stool eggs in order to confirm the species of schistosome. For every positive woman invited to participate, we also invited one uninfected woman to participate who presented for care in the same village on the same day. Women who consented to participate completed a structured interview involving demographic and clinical information, which was administered by the study nurse, and underwent additional testing for the larger study.

Each study participant provided 1–2 phone numbers, which were used by our study nurses to maintain contact with them for the duration of the study. Participants were asked to return for follow-up appointments at 3-month intervals for one year. At each follow-up, women again provided urine, stool, and blood samples for testing following the procedures described above. At the 6-month follow-up, women completed the survey administered at enrollment for a second time. At the 12-month follow-up, women completed the enrollment survey and also answered more detailed questions about water sources and exposure to lake water. All women who tested positive for schistosomiasis by urine, stool or serum CAA at any follow-up appointment were contacted by a nurse to return to clinic in order to receive directly observed treatment with praziquantel.

### Statistical analysis

Data was analyzed using Stata Version 13 (College Station, Texas). Demographic and clinical characteristics were quantified as number (percent) or median [interquartile range, or IQR] and compared using Wilcoxon rank-sum test for continuous variables, and chi-squared test for categorical variables. Fisher’s Exact test was used for categorical variables for which any category had less than 5 people. For factors that were significantly associated with recurrent or persistent schistosome infection using these initial tests, a multivariable logistic regression analysis was performed to determine which factors remained significantly associated with recurrent or persistent schistosome infection. Population at risk was defined as women non-infected with schistosomes. Cumulative incidence was calculated as the number of new cases divided by the total population at risk, and incidence rate in person-months was calculated as the number of new cases divided by the total months at-risk.

## Results

**Fig 1:**
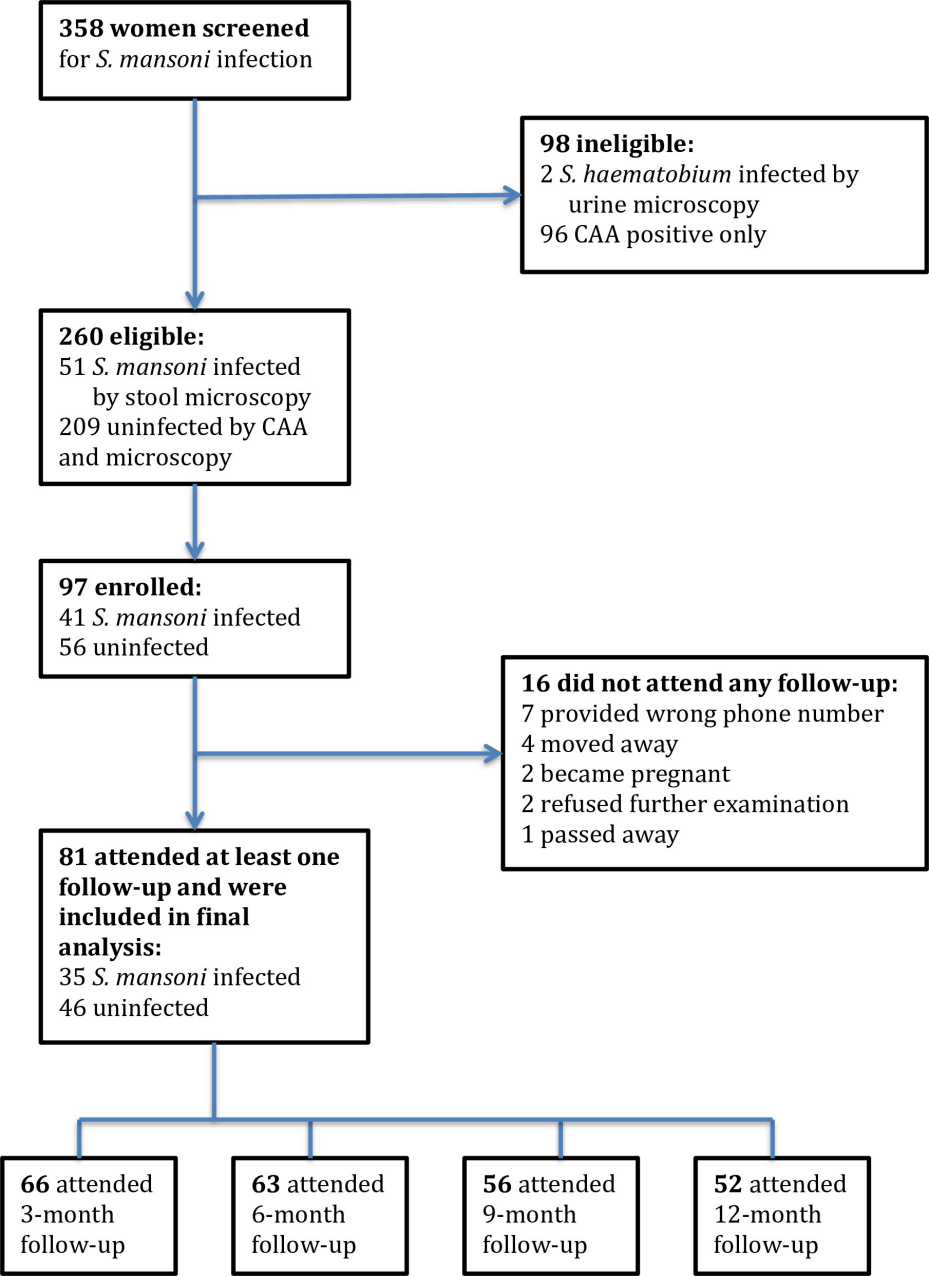
Flow chart of study screening and enrollment.

We screened a total of 358 women for schistosome infections by urine and stool microscopy and serum CAA testing. Of these, 51 women (14.2%) had *S*. *mansoni* eggs detected in stool and 2 women (0.5%) had *S*. *haematobium* eggs detected in urine (Fig 1). Serum CAA was positive in 141 women (39.4%).

41 of the 51 women who had *S*. *mansoni* eggs detected in stool returned to receive results and provided written informed consent to participate in the cohort study, along with an additional 56 women who came to receive their results on the same day and were completely negative for schistosome infection. Of these 97 women, 16 were not able to return for their first follow-up visit, as described in Fig 1. Ultimately, we analyzed data from 81 women who remained eligible for the larger study and returned for at least one follow-up visit: 35 with confirmed *S*. *mansoni* infection and 46 confirmed to be negative for schistosome infection.

Among the 35 women with *S*. *mansoni* infection, 31 were both CAA and stool microscopy positive and 4 were stool microscopy positive alone. The median stool eggs per gram was 24 [9.6–57.6] and the median CAA value was 1527.1 [216.2–10,000] pg/mL. At baseline, women with *S*. *mansoni* infection were younger at first pregnancy compared to uninfected women (p = 0.002). 3 women (8.6%) in the *S*. *mansoni* infected group, and 7 women (15.2%) in the uninfected group, were HIV-infected (p = 0.502) as expected due to decreased sensitivity of egg excretion in women with HIV [14]. There was also a trend towards more women with *S*. *mansoni* infection missing meals due to food insufficiency (66% versus 46%, p = 0.086). No other significant demographic or clinical differences were found between the two groups (Table 1).

**Table 1:** Demographic and clinical characteristics of women with and without *S*. *mansoni* infection at enrollment.

Characteristic (Median [IQR] or Number (Percent))	*S*. *mansoni* -infected (n = 35)	Schistosome-uninfected (n = 46)	p-value
**Age in years**	29 [23–38]	34 [27–42]	0.13
**Years of schooling**	7 [2–7]	7 [4–7]	0.43
**Number of children**	3 [1–5]	4 [2–6]	0.16
**Age at first pregnancy**	**18 [16–19]**	**19 [18–20]**	**0.007**
**Admits missing meals due to insufficient food in household in past month**	23 (66)	21 (46)	0.089
**HIV infected**	3 (8.6)	7 (15)	0.50
**Reports ever being treated for schistosomiasis prior to study enrollment**	6 (17)	15 (33)	0.12
Serum schistosome circulating anodic antigen (pg/ml)*	**1527.1 [216.2–10,000]**	**3.45 [0.0–8.5]**	N/A**
***S*. *mansoni* ova per 1 gram stool**	**24 [9.6–57.6]**	**0 [0–0]**	N/A**

* CAA < 30 pg/mL is negative.

** P-value is not applicable since these were criteria used to define the study groups.

**Fig 2:**
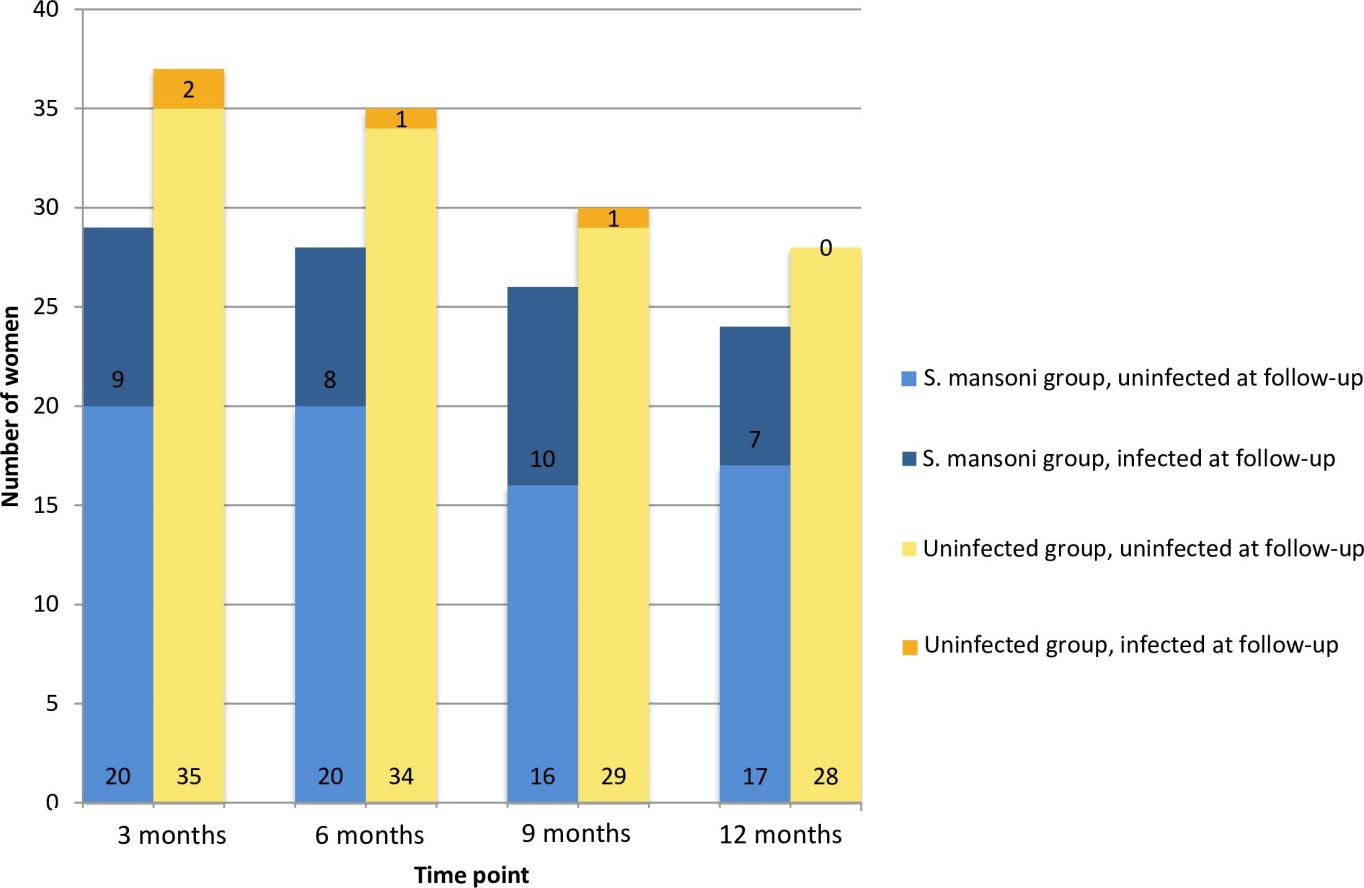
Number of women from each group who were seen at each follow-up time point, and their infection status. Blue bars represent women who were *S*. *mansoni*-infected at enrollment. Yellow bars represent women who were uninfected at enrollment. Darker color indicates the proportion of women who were *S*. *mansoni*-infected at the time point shown.

Fig 2 shows the number of women from each group (*S*. *mansoni-*infected or uninfected at enrollment) who were seen at each follow-up time point and the proportion who were schistosome-infected at that visit. 14 of the 81 women included in analysis were lost to follow-up at some point during the year. Of these, 4 did not want additional gynecologic examinations, 5 lost their phones and were no longer reachable, and 2 had family members who prohibited them from returning.

In total, 14 of the 35 women who had *S*. *mansoni* infections at baseline (40%) had infection again at some point during the 12-month follow-up period. The other 21 women (60%) cleared the infection after one treatment and did not become reinfected. Of the women who were *S*. *mansoni*-infected at baseline, 3 cleared the infection and then developed recurrent infection. 3 cleared the *S*. *mansoni* after multiple treatments, while 8 remained infected throughout the study period. Four women who were uninfected at baseline had developed incident *S*. *mansoni* infection by the 12-month follow-up, for a cumulative incidence of 8.7% and an incidence rate of 0.985 per 100 person-months among initially uninfected women throughout the entire study period.

**Fig 3:**
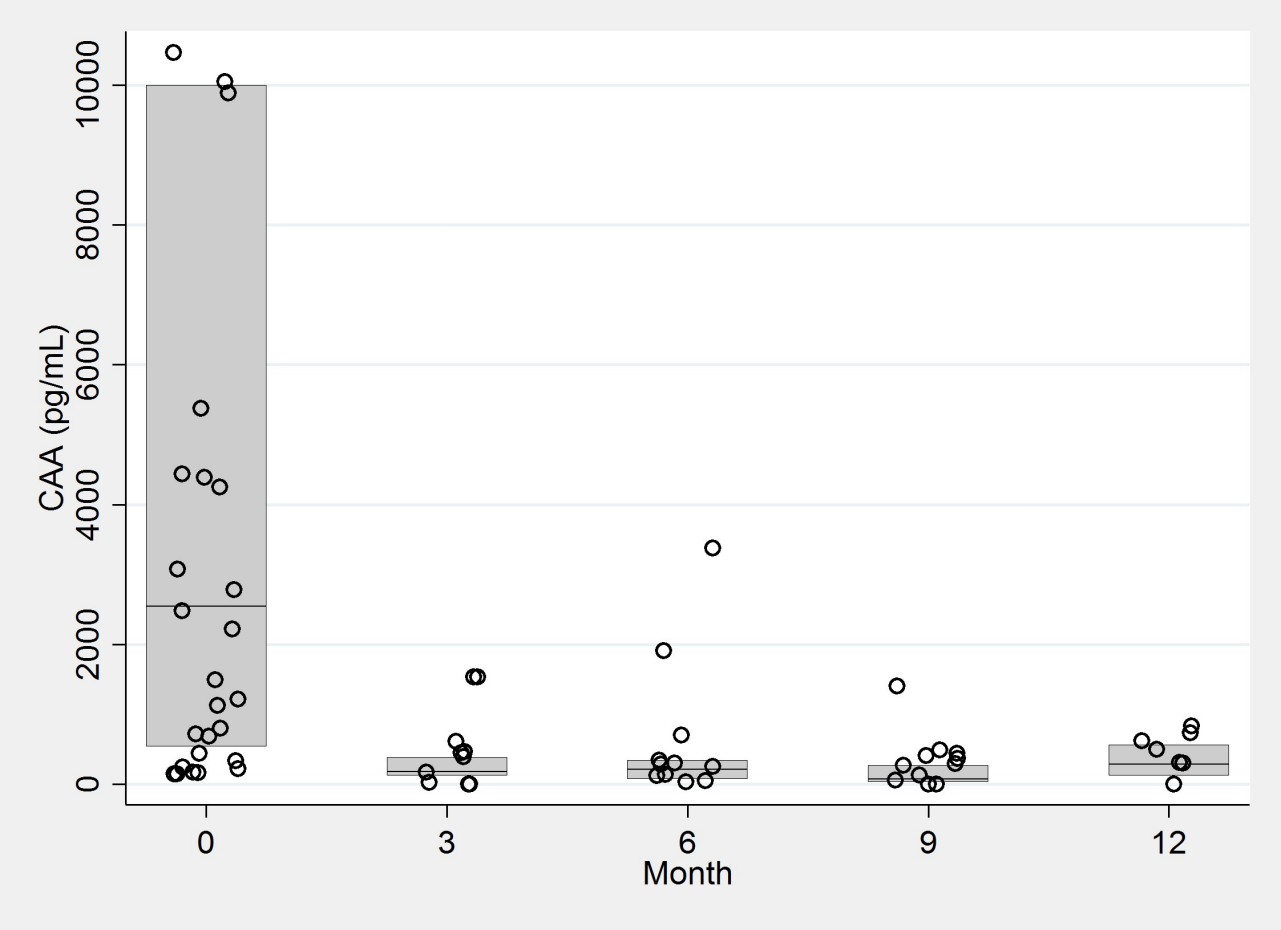
CAA values in women with *Schistosoma mansoni* infection at each time point. 6 data points with values > 12,000, all occurring at Month 0, were included in the derivation of this box plot but are not shown.

The majority of infections throughout the follow-up period were identified by elevated CAA levels only (CAA ≥ 30 pg/ml), with only 3 women found to be excreting eggs at any follow-up time point and an egg reduction rate of 98% from Month 0 to Month 3 [15]. The median CAA values at follow-up among those who were still CAA positive were significantly lower than the baseline CAA values at each subsequent time point (p = 0.001, Fig 3). No women were found to be excreting *S*. *haematobium* eggs in urine at any follow-up time point.

When compared with women who were schistosome-negative throughout all follow-ups, women with recurrent or incident infection during the study period were significantly younger (p = 0.032). They also had fewer children than women who remained uninfected (p = 0.003). Number of children remained significantly lower in women with schistosomiasis when controlling for age (p = 0.023). 8 out of 65 of the women without recurrent or incident infection (12.3%), and 2 out of 16 of those with recurrent or incident infection (12.5%) were HIV-infected (p = 1.00). No other significant demographic or clinical differences were found (Table 2).

**Table 2:** Demographic and clinical characteristics of women with and without recurrent or incident *S*. *mansoni* infection at any follow-up.

Characteristic (Median [IQR] or Number (Percent))	Recurrent* or incident infection at any follow-up (n = 16)	Schistosome-uninfected at all follow-ups (n = 65)	p-value
**Age in years**	**27 [22–33]**	**33 [27–41]**	**0.032**
**Years of schooling**	7 [1.5–7]	7 [4–7]	0.37
**Number of children**	**1.5 [1–3.5]**	**4 [2–6]**	**0.003**
**Age at first pregnancy**	17 [17–19]	18 [18–20]	0.13
**Admits missing meals due to insufficient food in household in past month**	10 (62)	34 (53)	0.51
**HIV-infected**	2 (12.5)	8 (12.3)	1.00
**Reports ever being treated for schistosomiasis prior to study enrollment**	4 (25.0)	17 (26.0)	0.92

*Recurrent infection was defined as the detection of eggs or serum CAA ≥ 30 pg/ml in a person who was previously schistosome-positive but most recently had been negative by both serum CAA and microscopy.

There was no significant difference in initial CAA level between women who were *S*. *mansoni* positive and successfully treated (1308 [161–10,000] pg/mL) compared to those with recurrent or persistent infection (1809 [586–7840], p = 0.587). Similarly, no difference was seen in the initial number of *S*. *mansoni* eggs per gram of stool in women who were successfully treated (24 [5–58]), compared to those with recurrent or persistent infection (34 [19–81], p = 0.423).

At enrollment, women with *S*. *mansoni* infection were significantly more likely to report the lake as their primary water source (20.0% versus 0.0%, p = 0.002), while women without schistosome infection were more likely to report piped water as their primary water source (17.1% versus 37.0%, p = 0.046). However, no difference in primary water source was seen when comparing women with recurrent or incident schistosome infection during the study to women who remained schistosome-negative throughout follow-up; lake water was used by 12.5% of women in the infected group and 7.7% of the schistosome-negative group (p = 0.620). Furthermore, no differences were noted between the groups in distance to primary water source (median of 30 [10–45] minutes walking in both groups) or number of water collection trips per week (median of 7 [7–14]).

A detailed survey about water contact was administered to women who were seen at the 12-month follow-up (n = 45). Women with recurrent or incident schistosomiasis were more likely to obtain drinking water from a well, while women who remained schistosome-negative were more likely to obtain drinking water from a pipe (p = 0.028). No difference was found in water sources used for bathing, cooking, washing clothes, or farming. Women were also asked whether they ever used lake water for any of these activities, and no significant difference was seen between the two groups in response to these questions (S1 Table).

Under 10% of women in each group reported fishing in the lake, and some also reported going into the lake for travel or fetching water in addition to the activities mentioned above. Women with recurrent or incident schistosomiasis were more likely to have gone swimming in the past 3 months (p = 0.023). Women in both groups lived a median of a 30 minute walking distance from the lake. Participants were also asked whether they were aware of the symptoms of schistosomiasis. 76% of responses correctly included “urinating blood”, “painful urination” and/or “pain in the stomach”, while 21% responded that they did not know. When asked whether they knew how to prevent schistosomiasis, 58% of respondents mentioned avoidance of drinking and bathing in dirty or standing water, wearing shoes or boots to enter the water, and boiling water before use. An incorrect response was given by 13%, and 29% responded that they did not know. There was no difference in correct knowledge of prevention strategies between women in either initial group (p = 0.406) or between women with recurrent or incident infection versus those who remained uninfected (p = 0.499).

## Discussion

Annual mass treatment of school-aged children with praziquantel is the current mainstay of schistosomiasis control. It has been hypothesized that protective immunity against schistosomes may develop over time, leading to a decreased prevalence and intensity of infection in adulthood despite continued exposure [16,17]. However, estimated rates of recurrence or persistence of infection after praziquantel treatment vary widely by region and population. By following up women with *S*. *mansoni* at 3-month intervals post-treatment, our study shows that an annual single-dose treatment with praziquantel is insufficient to clear the infection for as many as 40% of infected adult women living in this heavily endemic area.

In our cohort, the majority of women who were found to be schistosome-positive at follow-ups were women who were treated multiple times and were repeatedly CAA-positive. In these women the intensity of infection decreased after treatment, as evidenced by elimination of egg excretion and significant decreases in serum CAA, but CAA level remained above the threshold of positivity. This could have had several causes. First, praziquantel has decreased activity against juvenile worms [18] and therefore could lead to incomplete cure in recently infected people who harbored juvenile worms at the time of praziquantel treatment. Second, given that CAA becomes detectable 3–4 weeks post-infection [19], we were unable to determine whether women cleared infection but became re-infected, or whether the infection never cleared during the 3-month period between successive CAA measurements. Third, these findings could reflect technical or administrative imperfections or the presence of single-sex or non-egg producing infections [20].

Finally, it is possible that our findings could reflect incomplete clearance of infections, whether due to some level of resistance to praziquantel in schistosomes, or to varying pharmacokinetics of praziquantel or its synergy with host immunity in different persons [18]. Patterns of reduced treatment efficacy against *S*. *mansoni* have been observed in areas with heavy use of praziquantel. In Senegal, cure rates as low as 18–38% were seen with single-dose praziquantel, while treatment with oxamniquine yielded normal cure rates, leading to concern for developing drug resistance [21]. Reduced egg reduction rates have also been observed in Ugandan populations following multiple rounds of mass treatment [22]. In Egypt, *S*. *mansoni* isolated from patients who repeatedly failed treatment showed 3- to 5-fold lower sensitivity to praziquantel [23]. Although parasite resistance to praziquantel has not been demonstrated in Tanzania, we cannot rule this out as a contributing factor to our results.

Alternatively, the women who were repeatedly schistosome-positive may be more susceptible to reinfection than others for reasons related to individual immune response [24]. The women in our cohort who were persistently infected or reinfected were younger than those who were successfully treated. While this could be because younger women had more frequent or intense contact with contaminated water that was not captured by our survey, younger women also may have a less developed immune response due to less lifetime exposure to the parasite. Repeated treatment with praziquantel may further contribute to the development of immunity by increasing exposure to antigens from dying adult schistosome worms [25,26]. In our cohort, a similar proportion of women in each group reported previous treatment for schistosomiasis before the study, but we did not inquire how many times they had been treated. In contrast to some other reports, we did not find that intensity of initial infection by CAA or number of *S*. *mansoni* ova per gram, socioeconomic factors, or HIV infection were associated with treatment failure or increased risk of re-infection [27–29].

Only two women were found to be egg-positive after treatment at any follow-up. This could support the idea that the infections detected by CAA only were new infections rather than the result of unsuccessful treatment, since eggs would only be seen with an infection established for longer than about 6 weeks [30]. However, treatment with praziquantel is known to reduce schistosome egg production even in cases where it does not lead to cure [31]. Our results suggest that relying on microscopic examination for ova in this setting may lead us to overestimate the efficacy of praziquantel treatment in women, particularly given the documented sex differences in egg-to-CAA ratios between men and women [14].

In terms of parasite exposure during the study period, the majority of water-related risk factors were the same between the two groups, with the exception of swimming. This is usually considered an uncommon activity for adult women, but one-third of the infected women reported swimming in the lake in the past 3 months, so it may be more significant than often believed. Finally, our qualitative questions about how schistosomiasis can be prevented showed that many women are aware of precautions they should take against contaminated water. The women we surveyed are not necessarily representative of the rest of the community, as they have been educated about schistosomiasis multiple times through participating in the study. However, their responses indicate the difficulty of following water contact precautions in such a setting, even when education is provided.

Limitations of our study include a small sample size, which makes it difficult to control for confounders such as duration of infection, previous praziquantel treatments, or other medication use. Our conclusions are also limited by lack of information about other parasitic infections and immunologic data about the participants. The lack of post-treatment testing within 4–6 weeks after praziquantel to prove cure caused us to be unable to distinguish reinfection from persistent infection in some cases.

Our study identified an incidence of 8.7% among uninfected women over the course of one year, and recurrent or incompletely cleared infection affecting 40% of infected women. A single praziquantel treatment reduced intensity of schistosome infection, but was insufficient in providing stable parasite eradication in over a third of women. Further investigation into the efficacy of single-dose praziquantel in adults is warranted, especially as the interaction of schistosomiasis with HIV indicates that schistosomiasis control may have effects beyond control of the parasitic infection itself. Further analysis of data from this cohort will examine differences in cervical gene expression, immune environment, and microbiota in women with and without schistosomiasis, and the effects of treatment with praziquantel on these factors. Future studies may address effective treatment of schistosomiasis to reduce HIV risk in adult women in endemic areas.

## Supporting information

S1 Checklist STROBE checklist. (PDF)

S1 Table Further details about water contact in women who did and did not develop recurrent or incident S. mansoni infection. (PDF)
