# Supplementary material for: Insufficiency of annual praziquantel treatment to control Schistosoma mansoni infections in adult women: A longitudinal cohort study in rural Tanzania
Source: PLoS Negl Trop Dis. 2019 Nov 21;13(11):e0007844. doi: 10.1371/journal.pntd.0007844 (PMC6894890; doi:10.1371/journal.pntd.0007844)
Supplement: S1 Checklist — (PDF) [file pntd.0007844.s001.pdf]

S1 Checklist. STROBE Checklist

STROBE Statement—Checklist of items that should be included in reports of ***cohort studies***

	Item No	Recommendation
**Title and abstract**	1	(*a*) Indicate the study's design with a commonly used term in the title or the abstract<br>**Title page**
		(*b*) Provide in the abstract an informative and balanced summary of what was done and what was found<br>**Abstract, page 1**
**Introduction**		
Background/rationale	2	Explain the scientific background and rationale for the investigation being reported<br>**Introduction, paragraphs 1-2**
Objectives	3	State specific objectives, including any prespecified hypotheses<br>**Introduction, paragraph 3**
**Methods**		
Study design	4	Present key elements of study design early in the paper<br>**Materials and methods, paragraph 1**
Setting	5	Describe the setting, locations, and relevant dates, including periods of recruitment, exposure, follow-up, and data collection<br>**Results, paragraph 1**
Participants	6	(*a*) Give the eligibility criteria, and the sources and methods of selection of participants. Describe methods of follow-up<br>**Materials and methods, paragraph 3-5**
		(*b*) For matched studies, give matching criteria and number of exposed and unexposed<br>**Non-matched study**
Variables	7	Clearly define all outcomes, exposures, predictors, potential confounders, and effect modifiers. Give diagnostic criteria, if applicable<br>**Materials and Methods, paragraphs 1-4**
Data sources/ measurement	8*	For each variable of interest, give sources of data and details of methods of assessment (measurement). Describe comparability of assessment methods if there is more than one group<br>**Materials and Methods, paragraphs 3-4**
Bias	9	Describe any efforts to address potential sources of bias<br>**To address potential sampling bias, we recruited schistosome-negative controls from the same village and on the same day as the schistosome-positive women were enrolled, to minimise the differences between the two groups.**
Study size	10	Explain how the study size was arrived at<br>**The study size calculation was performed for the larger study for which this cohort was recruited, described in Materials and Methods, paragraph 2. . For this study, we included all women who were included in the larger study and attended at least one follow-up.**
Quantitative variables	11	Explain how quantitative variables were handled in the analyses. If applicable, describe which groupings were chosen and why<br>**Methods, paragraph 7**
Statistical methods	12	(*a*) Describe all statistical methods, including those used to control for confounding<br>**Materials and methods, paragraph 7**
		(*b*) Describe any methods used to examine subgroups and interactions

		(*c*) Explain how missing data were addressed<br>**Data in Tables 1 and 2 was complete. In Table 3, we included missing data in the category "No answer".**
		(*d*) If applicable, explain how loss to follow-up was addressed<br>**Loss to follow-up number and reasons are reported in Results, paragraph 4**
		(*e*) Describe any sensitivity analyses<br>**Not applicable**
**Results**		
Participants	13*	(a) Report numbers of individuals at each stage of study—eg numbers potentially eligible, examined for eligibility, confirmed eligible, included in the study, completing follow-up, and analysed<br>**Results, paragraph 1-2**
		(b) Give reasons for non-participation at each stage<br>**Results, paragraph 2/Fig 1**
		(c) Consider use of a flow diagram<br>**Results, Fig 1**
Descriptive data	14*	(a) Give characteristics of study participants (eg demographic, clinical, social) and information on exposures and potential confounders<br>**Results, paragraph 3/Table 1**
		(b) Indicate number of participants with missing data for each variable of interest<br>**Data in Tables 1 and 2 was complete. In Table 3, we included missing data in the category "No answer".**
		(c) Summarise follow-up time (eg, average and total amount)<br>**Results, paragraph 4/Fig 2**
Outcome data	15*	Report numbers of outcome events or summary measures over time<br>**Results, paragraphs 5-9, Fig 3, Table 2 and 3**
Main results	16	(*a*) Give unadjusted estimates and, if applicable, confounder-adjusted estimates and their precision (eg, 95% confidence interval). Make clear which confounders were adjusted for and why they were included<br>**Not applicable**
		(*b*) Report category boundaries when continuous variables were categorized<br>**Results, paragraph 5**
		(*c*) If relevant, consider translating estimates of relative risk into absolute risk for a meaningful time period<br>**Not applicable**
Other analyses	17	Report other analyses done—eg analyses of subgroups and interactions, and sensitivity analyses<br>**Not applicable**
**Discussion**		
Key results	18	Summarise key results with reference to study objectives<br>**Discussion, paragraph 1-2**
Limitations	19	Discuss limitations of the study, taking into account sources of potential bias or imprecision. Discuss both direction and magnitude of any potential bias<br>**Discussion, paragraph 6**
Interpretation	20	Give a cautious overall interpretation of results considering objectives, limitations, multiplicity of analyses, results from similar studies, and other relevant evidence<br>**Discussion, paragraph 2-5**
Generalisability	21	Discuss the generalisability (external validity) of the study results<br>**Discussion, paragraph 6**

Other information		
Funding	22	Give the source of funding and the role of the funders for the present study and, if applicable, for the original study on which the present article is based<br>**Entered into online submission form**

*Give information separately for exposed and unexposed groups.

Note: An Explanation and Elaboration article discusses each checklist item and gives methodological background and published examples of transparent reporting. The STROBE checklist is best used in conjunction with this article (freely available on the Web sites of PLoS Medicine at http://www.plosmedicine.org/, Annals of Internal Medicine at http://www.annals.org/, and Epidemiology at http://www.epidem.com/). Information on the STROBE Initiative is available at http://www.strobe-statement.org.
