# Supplementary material for: Insufficiency of annual praziquantel treatment to control Schistosoma mansoni infections in adult women: A longitudinal cohort study in rural Tanzania
Source: PLoS Negl Trop Dis. 2019 Nov 21;13(11):e0007844. doi: 10.1371/journal.pntd.0007844 (PMC6894890; doi:10.1371/journal.pntd.0007844)
Supplement: S1 Table — (PDF) [file pntd.0007844.s002.pdf]

S1 Table. Further details about water contact in women who did and did not develop recurrent or incident *S. mansoni* infection*

	**Recurrent or incident infection at any follow-up** (n=12)	**Schistosome-uninfected at all follow-ups** (n=37)	**p-value**
Primary drinking water source			**0.028**
Pipe	**4 (33.3)**	**24 (64.9)**	
Lake	**1 (8.3)**	**3 (8.1)**	
Well	**5 (41.7)**	**4 (10.8)**	
No answer	**2 (16.7)**	**6 (16.2)**	
Ever use lake water for drinking	5 (41.7)	8 (21.6)	0.26
Primary cooking water source			0.26
Pipe	5 (41.7)	23 (62.2)	
Lake	1 (8.3)	3 (8.1)	
Well	4 (33.3)	5 (13.5)	
No answer	2 (16.7)	6 (16.2)	
Ever use lake water for cooking	3 (25.0)	5 (13.5)	0.39
Primary bath water source			0.72
Pipe	5 (41.7)	17 (45.9)	
Lake	2 (16.7)	4 (10.8)	
Well	5 (41.7)	10 (27.0)	
No answer	0 (0.00)	6 (16.2)	
Ever use lake water to bathe	3 (25.0)	7 (18.9)	0.69
Primary laundry water source			0.70
Pipe	5 (41.7)	18 (48.6)	
Lake	2 (16.7)	4 (10.8)	
Well	4 (33.3)	8 (21.6)	
No answer	1 (8.3)	7 (18.9)	
Ever use lake water for laundry	1 (8.3)	9 (24.3)	0.41
Primary farm water source			0.43
Rainwater	5 (41.7)	18 (48.6)	
River	3 (25.0)	3 (8.1)	
Other	4 (33.3)	10 (27.0)	
No answer	0 (0.00)	6 (16.2)	
Ever use lake water for farming	1 (8.33)	2 (5.4)	1.0
Ever fish in lake	1 (8.33)	2 (5.4)	1.0
Ever swim in lake	5 (41.7)	7 (18.9)	0.14
Swam in lake in past 3 months	**3 (33.3)**	**1 (2.86)**	**0.023**
Entered lake for other reason in past 3 months**	6 (54.5)	9 (24.3)	0.074
For travel	3 (50.0)	5 (55.5)	
To wash dishes/clothes	2 (33.3)	3 (33.3)	
To fetch water	0 (0.00)	1 (11.1)	
To fish	0 (0.00)	1 (11.1)	
No answer	1 (16.6)	0 (0.00)	
Distance from lake (minutes walking)	30 [10-45]	30 [10-240]	0.62

*Survey administered to women seen at 12-month follow-up

**Multiple answers permitted
